# Switch to SGLT2 Inhibitors and Improved Endothelial Function in Diabetic Patients with Chronic Heart Failure

**DOI:** 10.1007/s10557-021-07254-3

**Published:** 2021-09-14

**Authors:** Michele Correale, Pietro Mazzeo, Adriana Mallardi, Alessandra Leopizzi, Lucia Tricarico, Martino Fortunato, Michele Magnesa, Salvatore Tucci, Pasquale Maiellaro, Giuseppe Pastore, Olga Lamacchia, Massimo Iacoviello, Matteo Di Biase, Natale Daniele Brunetti

**Affiliations:** 1 Ospedali Riuniti University Hospital, Foggia, Italy; 2 Department of Medical and Surgical Sciences, University of Foggia, Foggia, Italy

**Keywords:** Chronic heart failure, Type 2 diabetes mellitus, Sodium-glucose-cotransporter-2 inhibitors, SGLT2, Endothelial dysfunction, Gliflozins, Flow-mediated dilation

## Abstract

**Purpose:**

The use of sodium-glucose-cotransporter-type-2 inhibitors (SGLT2i) was associated in previous studies with an improved vascular function in non-human experimental models. We therefore sought to evaluate possible changes in endothelial function assessed by flow-mediated dilation (FMD) in patients with chronic heart failure (CHF) and type-2 diabetes mellitus (T2DM), switching from other oral hypoglycemic agents to SGLT2i in an observational study.

**Methods:**

Twenty-two consecutive outpatients with CHF and T2DM were enrolled after switching to SGLT2i therapy, and compared with 23 consecutive controls from the same registry comparable for principal clinical characteristics. In all patients, endothelial function was assessed by FMD at baseline and after 3 months of follow-up.

**Results:**

Three months of therapy with SGLT2i were associated with a statistically significant improvement in endothelial function (19.0 ± 5.7% vs 8.5 ± 4.1%, *p* < 0.0001); baseline levels of FMD were comparable between groups (p n.s.). Therapy with SGLT2i was significantly associated to improved FMD levels even at multivariable stepwise regression analysis (*p* < 0.001).

**Conclusions:**

Switch to SGLT2i in patients with CHF and T2DM was associated in an observational non-randomized study with an improved endothelial function.

**Supplementary Information:**

The online version contains supplementary material available at 10.1007/s10557-021-07254-3.

## Background

In the last decades, the prevalence of diabetes mellitus (DM) worldwide has almost doubled, from 4.7% in 1984 to 9.3% in 2019 [1]. Type-2 DM (T2DM) is a major risk factor for several cardiovascular (CV) conditions, including heart failure (HF) and endothelial dysfunction (ED).

Sodium-glucose-cotransporter-type-2 inhibitors (SGLT2i) represent a new class of anti-hyperglycemic agents for T2DM, which act as insulin independent to selectively inhibit renal glucose reabsorption, thereby increasing urinary glucose excretion. The EMPA-REG OUTCOME trial [2] was the first to demonstrate significant cardioprotective benefits in HF patients with empagliflozin, a SGLT2i. Its impressive 35% reduction in HF hospitalizations supported a possible role for SGLT2i among drugs for HF therapy, either with or without diabetes. This hypothesis was further confirmed in two other large randomized placebo-controlled trials, the CANVAS [3] with canagliflozin and the DECLARE-TIMI 58 [4] with dapagliflozin, and real-life data from the CVD-Real Study [5].

The use of SGLT2i was associated in previous studies with an improved vascular function in non-human experimental models [6–8]; this improvement may represent an important mechanism underlying the cardiovascular benefits of SGLT2i treatment. However, less is known on possible effect of gliflozins on endothelial function in humans. We therefore sought to evaluate possible changes in endothelial function assessed by flow-mediated dilation (FMD) in patients with CHF and T2DM switching from other oral hypoglycemic agents to SGLT2i in an observational study.

## Methods

Out of 250 patients enrolled in the Daunia Heart Failure Registry as reported elsewhere [9–11], 85 consecutive outpatients with CHF and T2DM were screened; 45 were enrolled and followed up between May 2019 and September 2020; 22 of them after switching to SGLT2i according to independent judgment of their referent endocrinologist in our University Hospital and recommendations of the position statement from Heart Failure Association of the European Society of Cardiology [12], the consensus report by the American Diabetes Association and the European Association for the Study of Diabetes [13] (in patients with HF receiving dual or multiple glucose-lowering medications, not including SGLT2i, a switch to an SGLT2i has been recommended), and 2019 ESC Guidelines on diabetes [14] (first-line treatment of DM in HF should include metformin and gliflozins). Exclusion criteria included HbA1c < 6.5%, eGFR < 60 mL/min/1.73 m^2^, age < 18 years, previous amputation surgery, recurrent urinary tract infections, LVEF > 50%, and changes in the antidiabetic treatment during the study. Patients with other diabetes-related comorbidities (retinopathy, nephropathy …) were excluded.

Twenty-two consecutive patients were therefore treated with SGLT2i and compared with 23 consecutive controls from the same registry comparable for principal clinical characteristics who did not switch to therapy with gliflozins.

In all patients, endothelial function was assessed by FMD at baseline and after 3 months of follow-up. Medical history, heart rate, systolic blood pressure, body mass index, NYHA functional class, and medications were recorded. All patients underwent blood analysis and ECG in an ambulatory setting, under resting conditions at baseline and after 3 months of therapy with SGLT2i. Glycated hemoglobin (HbA1c), high-sensitivity C-reactive protein (CRP), and NTproBNP levels were assayed. Circulating levels of CRP were assessed on venous blood samples drawn from the antecubital vein, using an ethylenediamine-tetra-acetic acid (EDTA) test tube; high-sensitivity CRP was quantified by a highly sensitive immunoassay technique.

### Endothelial Function

FMD of the brachial artery was assessed under standardized condition, according to the “Brachial Artery Reactivity Task Force’s guidelines” [15]. The patients were fasting and avoided exercise for at least 8 h before the exam as well as nicotine, exciting substances like chocolate or coffee/tea for at least 4 h and all medications affecting vascular tone or cardiac output for four half-lives. A preliminary scan explored the anatomy and identified landmarks, paying attention to the presence of calcifications, arterial tortuosity, and atherosclerotic plaque. The brachial artery was evaluated in a long axis projection between 5 and 10 cm above the elbow. All patients were evaluated by the same operator to minimize the bias. A high-resolution US device Philips EPIQ 7C ultrasound system with vascular software, a 7.5–12-MHz linear array transducer, and an ECG-gated image capture system for calculating the brachial artery diameter in real time by analyzing B mode US images were used. The arm was positioned comfortably thanks to a stereotactic probe-holding device. With the subject in supine position for at least 10 min, a sphygmomanometer cuff was placed in the distal site to the artery, in cases of a humeral artery on the forearm. After 1 min of flow image baseline acquisition, the artery was occluded by inflating the cuff to a suprasystolic pressure for 5 min (typically at least 50 mmHg above systolic pressure).

Reactive hyperemia was evaluated as the ratio of the change in diameter (maximal dilatation after deflation baseline) divided by the baseline value, which corresponds to the maximum FMD recovery value. FMD was analyzed as the percentage increase in brachial artery diameter after the application of a pressure stimulus, as reported elsewhere [16].

### Statistical Analysis

Continuous variables were expressed as mean ± standard deviation and compared with Student’s *t*-test, and categorical variables as percentages and compared with *χ*^2^ test. Mean values were compared with Student’s *t*-test for variables with a normal distribution or with the Mann–Whitney non-parametric *U* test for variables with a non-normal distribution. Repeated measures were compared with Student’s *t*-test for paired samples, and Wilcoxon’s test and ANOVA for repeated measured as required. Linear correlations were determined by measuring the Pearson correlation coefficient. Multivariable stepwise regression analysis was used to assess possible bias of confounders. A *p* < 0.05 was considered statistically significant.

### Sample Sizing

On the base of prior data on FMD changes [17], an expected FMD change of at least 5% over time, an 80% power, and *α* of 0.05, groups of at least 11 subjects were required for the analysis.

## Results

Population characteristics are given in Table 1; 81% of patients were treated with beta-blockers, and 86% with ACE-inhibitors/angiotensin receptor blockers/angiotensin neprilysin inhibitors.

**Table 1:** Population’s characteristics

Variables	SGLT2 inhibitors		Other antidiabetic drugs		*p*-value
	Mean ± SD	%	Mean ± SD	%	
Age (years)	66 ± 9		71 ± 9		0.0693
Male (%)		91%		70%	0.0765
COPD (%)		14%		26%	0.3074
Hypertension		77%		74%	0.7988
Obesity (BMI > 30)		36%		35%	0.9143
NYHA class	2.1 ± 0.5		2.2 ± 0.7		0.1358
Years of diabetes	9 ± 7		9 ± 4		0.7439
Systolic blood pressure (mmHg)	118 ± 28		112 ± 16		0.3756
Creatinine (mg/dl)	1.0 ± 0.2		1.1 ± 0.2		0.2730
NT-pro-BNP (pg/ml)	1595 ± 2411		638 ± 607		0.0730
HbA1c (%)	8 ± 1		8 ± 1		0.9879
Left ventricular ejection fraction (%)	41 ± 7		40 ± 7		0.8254
ACEi/ARB/sacubitril-valsartan (%)		86%		87%	0.9073
Beta-blockers (%)		91%		81%	0.3290
Mineralcorticoid receptor antagonist (%)		62%		35%	0.0750
Loop diuretics (%)		86%		83%	0.7846
Ivabradine (%)		14%		4%	0.2624
Digoxin (%)		10%		4%	0.5076

Twenty-two patients were treated with SGLT2 inhibitors and compared with twenty-three controls comparable for principal clinical characteristics. Of 22 patients treated with SGLT2i, 64% (14) received empagliflozin, 18% (4) dapagliflozin, and 18% (4) canagliflozin; 3 were also treated with both insulin and oral antidiabetics (14%), 8 with insulin (36%), and 9 with OAD (41%). Of 23 patients not receiving SGLT2i, 2 were treated with both insulin and OAD (8.7%), 5 with insulin (21.7%), and 16 with OAD (69.6%). Data on OAD are given in Supplementary Fig. 1 (S1).

**Fig. 1:**
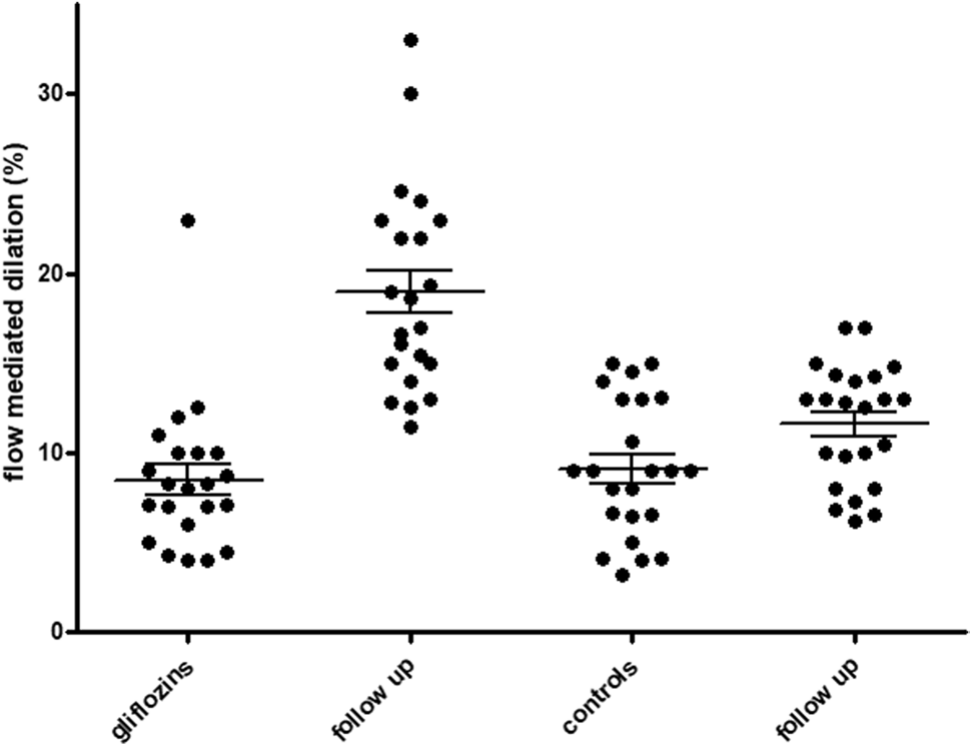
Stress-rest radial artery diameter difference (%) derived by flow-mediated dilation between patients in therapy with SGLT2 inhibitors and patients with other antidiabetic drugs

After a 3-month follow-up, the patients who started therapy with SGLT2i showed a statistically significant improvement in endothelial function (19.0 ± 5.7% vs 8.5 ± 4.1%, *p* < 0.0001, ANOVA *p* vs controls < 0.001, Fig. 1); baseline levels of FMD were comparable between groups (p n.s.).

Treatment with SGLT2i was also associated with statistically significant reductions in glycated hemoglobin levels (7.7 ± 1.0% vs 8.2 ± 1.2%, *p* < 0.01) and non-significant reductions in CRP (2.0 ± 2.2 vs 3.0 ± 3.6 mg/dl, p n.s.) and NTproBNP levels (581.6 ± 564.5 vs 1609.3 ± 2543.3, *p* 0.09).

**Fig. 2:**
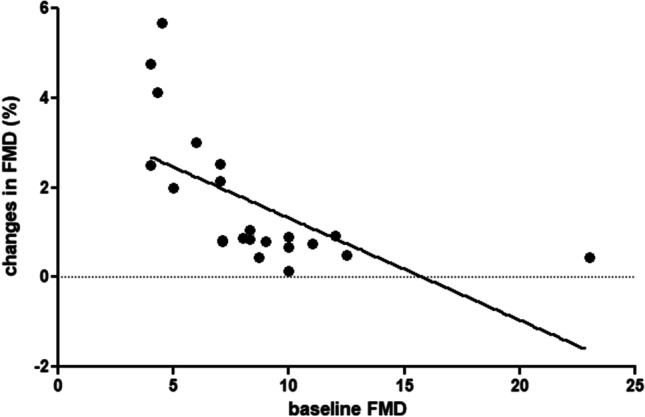
Correlation between baseline FMD values and changes in FMD (in percent, *p* < 0.05)

Changes in FMD values were not proportional to changes in NTproBNP, CRP, and glycated hemoglobin levels at univariate analysis, but were related to baseline FMD values (*r* =  − 0.62, *p* < 0.05, Fig. 2). Changes in FMD values were not proportional to baseline glycated hemoglobin levels.

Therapy with SGLT2i was significantly correlated to improved FMD levels even at multivariable stepwise regression analysis in a model including age, gender, baseline FMD levels, LVEF, EDV, NTproBNP, CRP, HbA1c values, changes in percent of NTproBNP, CRP, and HbA1c (*p* < 0.001) (Table 2).

**Table 2:** Predictors of improved flow-mediated dilation at multivariable stepwise regression analysis

	*b*	Std. Err	*p*-value
Baseline flow-mediated dilation levels	− 0.15	0.04	0.0034
Use of SGLT2 inhibitors	1.33	0.34	0.0009
Change in glycated hemoglobin levels	− 7.30	2.70	0.0140
Change in C-reactive protein levels	0.76	0.34	0.0373
Baseline C-reactive protein levels	− 0.16	0.07	0.0447

## Discussion

To the best of our knowledge, this one the first studies showing an improved endothelial function after therapy with gliflozins in patients with CHF and T2DM. The improvement in endothelial function does not seem to likewise correlate with changes in NTproBNP, CRP, and glycated hemoglobin levels, suggesting an independent effect of gliflozins on endothelial function beyond glucose-lowering effects.

Impaired endothelial function plays the pivotal role in the pathophysiology of atherogenesis and is usually accompanied by increased oxidative stress and inflammatory responses. ED may also play an important role in the pathogenesis of HF. The interplay between CHF, inflammation, and ED is extremely complex and multifaceted [18]. CHF is characterized by an inflammatory activation [19] and ED, partly secondary to systemic inflammation. Oxidative stress has been shown to play an important role in the pathophysiology of cardiac remodeling and HF [20].

Recent animal studies have demonstrated that treatment with SGLT2i can reduce oxidative stress and the inflammation processes. The inhibition of SGLT2 by phlorizin prevents the hyperglycemia and oxidative stress in kidney of diabetic rats [21]. Empagliflozin significantly ameliorated in mice the impairment of vascular dilating function, with attenuation of oxidative stress in cardiovascular tissue [22]. In high-fat diet and streptozotocin-nicotinamide-induced type 2 diabetic mice which exhibit impaired insulin secretion, insulin resistance, hyperlipidemia, hepatic steatosis, and obesity, ipragliflozin reduced plasma and liver levels of oxidative stress biomarkers and inflammatory markers (interleukin 6, tumor necrosis factor α, monocyte chemotactic protein-1, and CRP) [23].

The use of SGLT2i is associated in previous studies with an improved vascular function in animal models. Empagliflozin improved diabetes-induced vascular dysfunction in the streptozotocin diabetes rat model by interfering with oxidative stress and glucotoxicity [24]. Glycemic control with ipragliflozin ameliorated endothelial dysfunction in streptozotocin-induced diabetic mouse [25].

Our results are in line with several previous works in experimental models. Juni et al. demonstrated that therapy with empagliflozin may provide beneficial effects on cardiac microvascular endothelial cells by reducing mithocondrial ROS production and cytoplasmatic ROS accumulation, which led to restoration of endothelial NO bioavailability [26]. Shigiyama et al. [27] reported that 16 weeks of treatment with dapagliflozin, in patients with a short duration of T2DM and no history of atherosclerotic CVD, improved endothelial function measured by flow-mediated vasodilation, compared with that associated with an increased dose of metformin. Solini et al. [28] also reported that 2 days of treatment with dapagliflozin acutely improved endothelial function in T2DM patients at low risk of CV events.

However, in the EMBLEM trial [29], a multicenter, placebo-controlled, double-blind, randomized trial designed to evaluate the effect of empagliflozin on endothelial function as assessed by reactive hyperemia-peripheral arterial tonometry (RH-PAT) in patients with T2DM and established CVD, 24 weeks of treatment did not affect endothelial function compared with placebo. The study did not include a detailed evaluation of responders and non-responders to empagliflozin therapy. In a secondary and exploratory analysis of the study by Tanaka et al. [30] empagliflozin treatment for 24 weeks had fewer direct effects on vascular function, at least in patients with T2DM and established CVD. It is quite likely that population bias might have influenced the results of this trial; several T2DM patients had a history of established CVD, including or HF or coronary artery disease or stroke or peripheral artery disease or the presence of coronary artery stenosis (≥ 50%).

Recently, rationale and design of an investigator-initiated, multicenter, prospective open-label, randomized trial to evaluate the effect of ipragliflozin on endothelial dysfunction in T2DM and chronic kidney disease (the PROCEED trial) [31] and of a prospective, single-center, investigator-blinded, open-label, randomized clinical trial (empagliflozin 25 mg/day alone or empagliflozin 25 mg/day plus evolocumab 140 mg every 2 weeks in addition to optimal medical care) to evaluate the add-on effect of PCSK9i on endothelial function of T2DM patients under regular use of empagliflozin [32] have been published.

Results from ADDENDA-BHS2 study (a prospective, single-center, active-controlled, open, randomized trial, where ninety-eight participants were randomized to either dapagliflozin 10 mg/day or glibenclamide 5 mg/day on top of metformin) demonstrated that dapagliflozin improved micro- and macrovascular endothelial function compared to glibenclamide (rest FMD changed by + 3.3(8.2)% and − 1.2(7.5)% for the dapagliflozin and glibenclamide arms, respectively (*p* = 0.0001)), regardless of glycemic control in patients with T2DM and subclinical carotid atherosclerotic disease [33]. Several hypotheses have been proposed to explain cardioprotective effects of SGLT2 inhibitors [34, 35]. SGLT2 inhibitors are hypothesized to increase natriuresis, with unique properties leading to a reduction in preload and myocardial stretch (the diuretic hypothesis); exert many anti-inflammatory effects (the anti-inflammatory effects hypothesis); act through the angiotensin II type II receptors leading to vasodilation (the RAAS hypothesis); and exhibit also inhibitory action possibly resulting in an attenuation of oxidative stress (the SGLT1 inhibition hypothesis) [36]. Irace et al. demonstrated that, through osmotic diuresis, empagliflozin may increases blood viscosity and, consequently, shear stress, which is the major hemodynamic force acting on the endothelial surface and promoting the synthesis of vasoactive and athero-protective substances [37, 38]. Dapagliflozin induces vasodilation via the activation of Kv channels and protein kinase G, and is independent of other K + channels, Ca2 + channels, intracellular Ca2 + , and the endothelium [39].

Sympatholysis, i.e., reduction in sympathetic nervous system (SNS) activity, could be a potential mechanism for the apparent endothelial benefits of the SGLT2i switch in the patients affected by HF with T2DM. Probably, SGLT2is alleviate the renal stress responsible for sympathetic activation [40]. Despite increasing experimental and clinical data [41] demonstrated a reduction in SNS activity, the mechanisms linking SGLT2 and SNS need of further investigation. However, the failure to increase heart rate despite reductions in blood pressure and plasma volume may suggest a decrease of SNS activity in patients affected by HF with or without T2DM in treatment with gliflozins.

Randomized adequately powered studies are warranted to confirm vasoactive effect of SGLT2i preliminary shown in small observational studies.

## Conclusions

Switch to SGLT2i in patients with CHF and T2DM was associated in an observational non-randomized study with an improved endothelial function.

### Limitations

Main limitation of the study is the small number of patients enrolled and the observational nature of the study; these preliminary results need to be confirmed in properly powered multicentric and randomized studies.

No data are available on plasma levels of nitrite/nitrate and or endothelin, which would be of great interest in order to clarify possible pathways of interaction.

The study is underpowered to consider possible difference between groups of borderline significance (see mineralocorticoids receptor antagonists).

## Supplementary Information

Below is the link to the electronic supplementary material.

Supplementary file1 (DOCX 564 KB)

## Data Availability

Data are available on reasonable request.
