# Supplementary figures and images for: Switch to SGLT2 Inhibitors and Improved Endothelial Function in Diabetic Patients with Chronic Heart Failure
Source: Cardiovasc Drugs Ther. 2021 Sep 14;36(6):1157–64. doi: 10.1007/s10557-021-07254-3 (PMC9652233; doi:10.1007/s10557-021-07254-3)

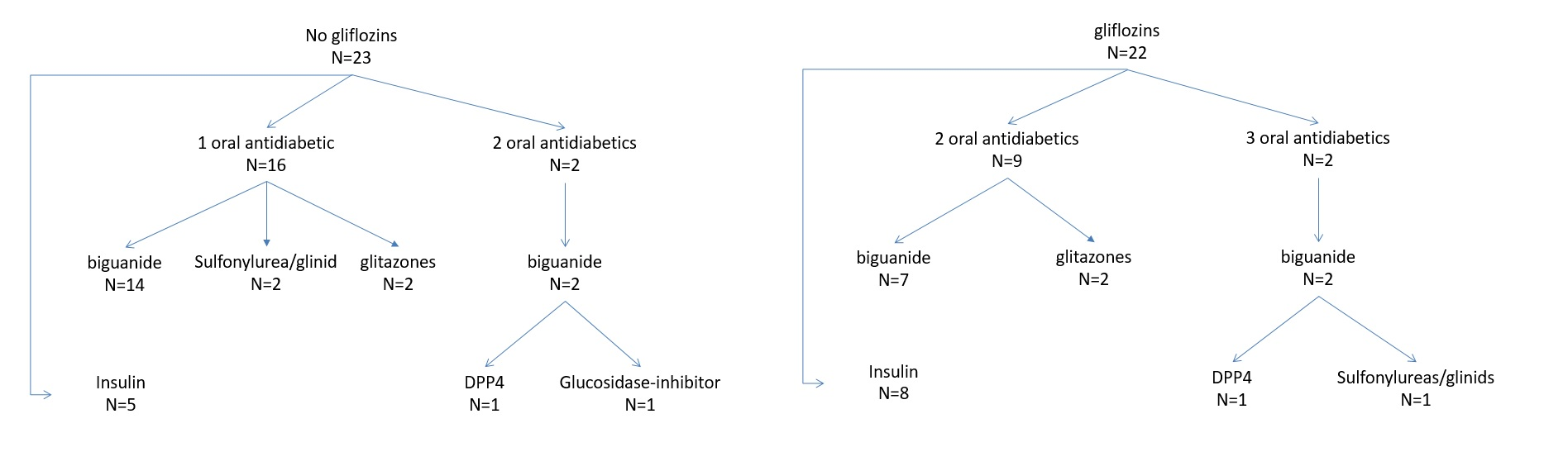


Supplementary Figure 1 (S1). Use of additional oral antidiabetics.

Supplement: Supplementary file 1 — Supplementary file1 (DOCX 564 KB) [file 10557_2021_7254_MOESM1_ESM.docx]
